# Targeted delivery of antitubercular drugs using glucan lipid particles

**DOI:** 10.1128/spectrum.02744-24

**Published:** 2025-02-06

**Authors:** Eleni Jaecklein, Kadamba Papavinasasundaram, Gary R. Ostroff, Christopher Sassetti, Ernesto R. Soto

**Affiliations:** 1 Department of Microbiology and Physiological Systems, University of Massachusetts Chan Medical School, Worcester, USA; 2 Program in Molecular Medicine, University of Massachusetts Chan Medical School, Worcester, USA; CNRS - University of Toulouse, France

**Keywords:** glucan lipid particles, tuberculosis, drug delivery

## Abstract

Simpler, safer, and faster chemotherapeutic regimens for tuberculosis and other respiratory mycobacterial infections are an urgent need. Many current therapies suffer from suboptimal drug exposure and dose-limiting systemic adverse effects, challenges that could be addressed via controlled delivery of drugs to the primary site of infection. We sought to address this need by designing a flexible formulation platform that targets drugs to the lung macrophages that concentrate at infectious foci. Our approach is based on an encapsulation strategy in which drugs or specifically designed prodrugs are captured in the hydrophobic core of a glucan-lipid particle (GLP). We show chemically diverse antimycobacterial drugs can be efficiently and stably encapsulated within GLP and that these microparticles can be engineered to release drugs upon low pH or reducing conditions that occur upon phagocytosis by macrophages. Encapsulated formulations of clofazimine, isoniazid, and linezolid retain activity against intracellular *Mycobacterium tuberculosis* (Mtb) in an *ex vivo* model, demonstrating efficient drug delivery and release. Intranasal administration of GLP-clofazimine to *Mtb*-infected mice effectively concentrates the drug in the lung and reduces bacterial burden, whereas GLP-delivered linezolid was systemically distributed and failed to inhibit bacterial growth in the lung. This work establishes GLPs as a promising platform for targeted antibiotic delivery to the lung and also illustrates pharmacokinetic parameters that must be considered in future development.

**IMPORTANCE:**

Tuberculosis (TB) causes an estimated 10.8 million cases each year and remains one of the leading causes of infectious death. Effective treatment is complicated due to the lengthy drug regimen required to prevent relapse and treatment failure. A primary challenge is delivering drugs effectively to lung granulomas, where TB bacteria can persist. Here, we developed yeast-derived glucan lipid microparticles (GLPs) as a novel delivery system to efficiently encapsulate and deliver TB drugs directly to lung tissue via intranasal administration. Of the formulations evaluated, GLP-encapsulated clofazimine achieved increased lung drug levels and reduced bacterial burden in TB-infected mice. The use of GLPs offers a promising approach to improve TB treatment by enabling targeted drug delivery to infection sites within the lungs.

## INTRODUCTION

The World Health Organization (WHO) aims to reduce tuberculosis (TB) incidence by 80% (from 2015) by 2030 (1). Although TB incidence and deaths have decreased since 2015, the rate of decline is far behind the targeted milestones with an estimated 10.8 million individuals developing TB and 1.25 million succumbing to the disease in 2023 (1). Standard treatment for TB is a lengthy, 6- to 9-month course of multiple antibiotics. Although this current regimen is generally effective against drug-susceptible TB, poor treatment adherence and the emergence of drug-resistant strains lead to suboptimal efficacy on a larger scale (2, 3). Multidrug-resistant TB (MDR-TB) and extensively drug-resistant TB (XDR-TB) require longer treatment with second-line antibiotics that often have toxic side effects. Although recent clinical trials show that the duration of therapy can be reduced for many patients, treatment remains challenging due to adverse drug effects and implementation difficulties (3–6). Thus, there is an urgent need to discover and optimize regimens that are shorter, safer, and more accessible (2). Although there are new drugs in the development pipeline, modifying existing drugs to be safer and more efficacious may be a faster, more cost-effective solution (7).

A key challenge for treating TB is achieving and maintaining inhibitory concentrations of drugs at the primary site of infection in the lung (8). The hallmark histological feature of TB is the granuloma, and drug penetration into these sites is highly heterogeneous and dependent on the specific drug as well as the lesion structure (9). Prolonged bacterial exposure to subtherapeutic levels of the drug contributes to treatment failure, the development of resistance, and disease relapse (9). Delivering antibiotics directly to the lungs could serve to increase local drug concentrations while limiting systemic exposure that leads to many adverse TB drug effects such as hepatotoxicity, neuropathy, discoloration, and bone marrow suppression (5, 10). Additionally, inhalable variations of injectable drugs for the treatment of MDR and XDR-TB, such as aminoglycosides, have clear advantages for patient comfort and compliance. Previous work has shown that inhalable versions of both first- and second-line TB therapeutics can be efficacious when tested in models of infection (11–17). In fact, a phase-I clinical trial of inhalable capreomycin, an injectable aminoglycoside used to treat MDR-TB, showed that the drug was well tolerated and achieved concentrations in the plasma that exceeded the Minimum Inhibitory Concentration (MIC) (18). Beyond delivering drugs directly to the lungs, ensuring they reach the site of infection is critical to their efficacy. The classical TB granuloma is a macrophage-rich cellular structure surrounding a necrotic core. *Mycobacterium tuberculosis* (*Mtb*) resides both in this necrotic material and inside the surrounding macrophages. Concentrating antibiotics on lung macrophages via aerosolized particles has been shown to increase drug concentration in that cell population (17, 19). Infection models in zebrafish, mice, and nonhuman primates indicate that there is a large degree of cellular movement within and around the granuloma (20–22). Therefore, we hypothesized that targeting antibiotics to macrophages may be an efficient method of concentrating drugs throughout inflammatory sites, such as granuloma, where these cells accumulate.

Glucan particles (GPs) can be used to target diverse payloads to macrophages and other phagocytic cells (23–26). GPs are hollow spheres (2–4 µm in diameter) derived from Baker’s yeast, *Saccharomyces cerevisiae,* that can absorb up to 10× their weight in water allowing for diffusion of water-soluble compounds through the pores of the swollen particle. The β−1,3 glucans on the surface of the GPs are recognized by cell surface receptors like Dectin-1 and Complement receptor 3 (CR3), which are highly expressed on macrophages and other phagocytic cells (27, 28). The GPs are engulfed via receptor-mediated phagocytosis, and the payload can be designed for preferential intracellular release (23). GPs are amenable to aerosolization, which, in combination with their affinity for macrophages, makes them an attractive vehicle for delivering antitubercular therapies (24–26). However, GPs are not suitable for the encapsulation of chemically diverse TB drugs. Hydrophobic compounds are encapsulated at low efficiency (29), and hydrophilic compounds are difficult to stably capture.

Here, we utilize glucan lipid particles (GLP) to efficiently load water-insoluble payloads (30). By modifying the GP preparation procedure, we created particles that retain non-saponifiable lipids in the membrane (e.g., cholesterol and ergosterol). The retention of some lipid components in GLPs allows for efficient loading of hydrophobic payloads. In this study, we used GLPs to stably encapsulate high concentrations of the hydrophobic antibiotics rifabutin (RFB) and clofazimine (CFZ) and water-insoluble pro-drug forms of the more hydrophilic drugs isoniazid (INH) and linezolid (LZD). These formulations were designed preferentially to release active drugs after internalization by the macrophage, and we show that encapsulated INH, LZD, and CFZ maintained antibacterial activity in *Mtb*-infected macrophages. Intranasally delivered GLP-CFZ formulations reduced bacterial burden in mice and increased the portion of the drug concentrated in the lung in comparison to orally delivered CFZ, potentially providing a formulation that would reduce stigmatizing skin discoloration that results from systemic exposure to this drug. These experiments demonstrate the versatility of GLPs and their potential as an inhalable drug delivery platform for the treatment of mycobacterial infections.

## RESULTS

### GLP encapsulation approaches for hydrophilic TB drugs

**Fig 1:**
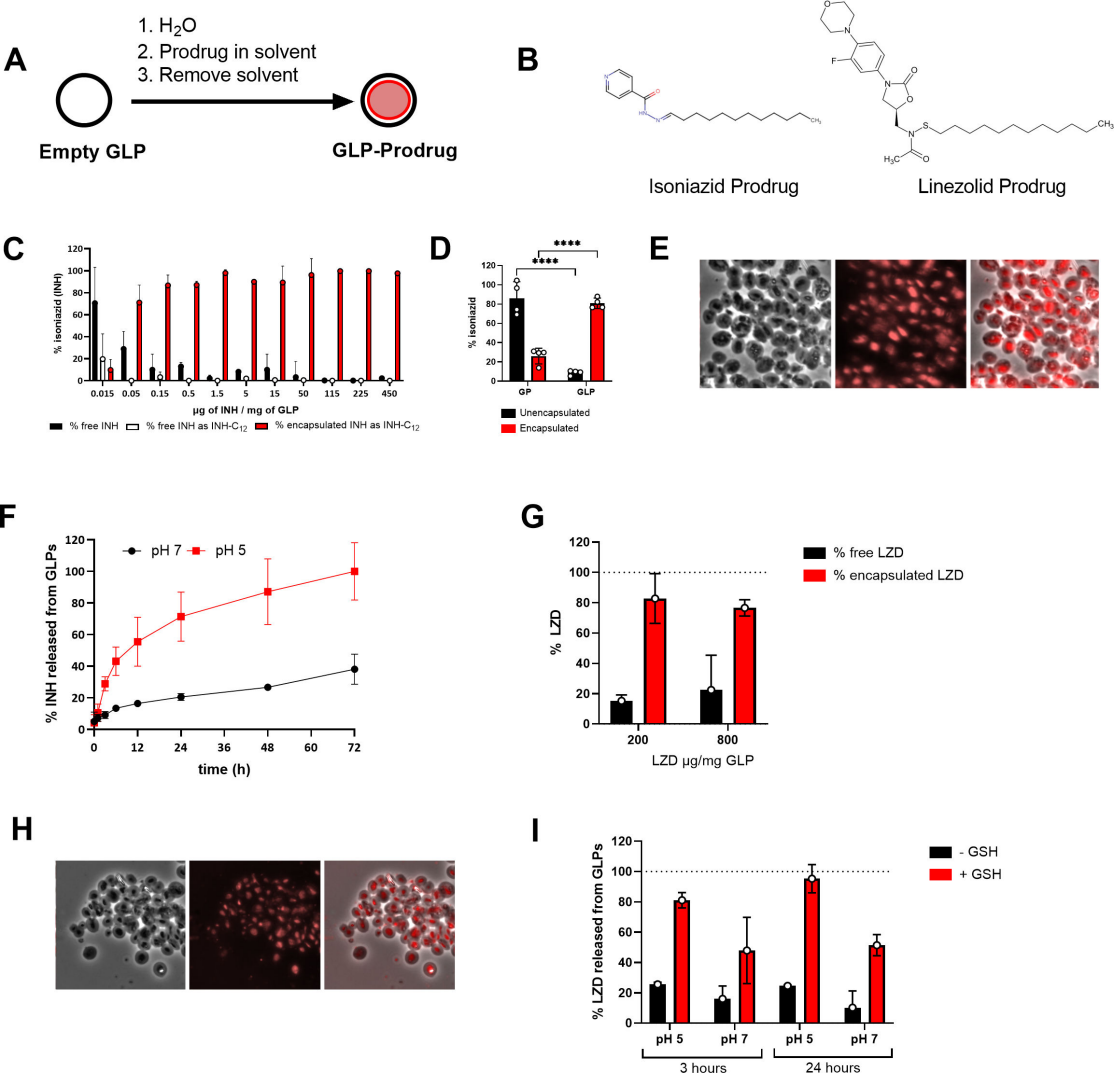
Hydrophobic INH and LZD prodrugs are stably encapsulated in GLPs. (A) Schematic of loading GLPs with prodrug. (**B**) Chemical structures of INH and LZD prodrugs. (**C**) Encapsulation efficiency of INH-prodrug in GLPs measured by HPLC. (**D**) Quantification of free versus encapsulated INH-prodrug in GPs and GLPs using HPLC. (**E**) Micrographs showing INH-prodrug encapsulated in GLPs using Nile red staining. (**F**) INH release from GLP samples diluted at 50 µg INH/mL in 50 mM acetate buffer (pH 5) or 50 mM phosphate buffer (pH 7). Samples were incubated at 37ᵒC. Data shown correspond to GLP-INH prodrug samples prepared at 115 µg INH/mg GLP. (**G**) Encapsulation efficiency of LZD prodrug at two target LZD/GLP weight ratios. (**H**) Micrographs showing LZD-prodrug encapsulated in GLPs using Nile red staining on the alkyl chain. (**I**) LZD release from GLP samples diluted at 500 µg LZD/mL in (pH 5) or 50 mM phosphate buffer (pH 7) with or without 10 mM glutathione (GSH). Samples were incubated at 37ᵒC for the indicated times. Nile red microscopy exposure time: 50 ms, rhodamine channel. Data depicted are single experiments that are representative of three independent studies.

INH and LZD are critical components of first- and second-line TB therapy, respectively. Both drugs are water-soluble, making them difficult to trap in glucan particles. We created hydrophobic prodrug constructs of both INH and LZD that are more amenable for encapsulation in GLP (31, 32) (Fig. 1A). The prodrug constructs are (i) water-insoluble allowing for loading and retention inside the hydrophobic hollow cavity of GLPs, (ii) stable at neutral pH, and (iii) utilize biodegradable linkers that are susceptible to chemical hydrolysis when exposed to low pH or reducing conditions (31–33) encountered after particle uptake by macrophages, which allow for controlled release.

A Schiff base prodrug of INH with an acid-labile linker (Fig. 1B) was synthesized by reaction of INH with dodecanal. The prodrug was loaded in GLPs (Fig. 1A), and encapsulation efficiency was quantified over a range of loading concentrations. This prodrug was encapsulated with >80% efficiency at concentrations higher than 0.15 µg INH/mg GLP (Fig. 1C; Table 1). At the two lowest INH/GLP ratios, the concentration of INH prodrug in the GLP prodrug samples was below the water solubility of the INH-prodrug in water resulting in prodrug release and hydrolysis. The encapsulation efficiency was much lower in GP, compared with GLP, demonstrating the importance of the hydrophobic character of the GLP (Fig. 1D). To ensure the prodrug was loaded inside the GLPs, we stained the hydrophobic C_12_ chain of the prodrug with Nile red and used fluorescence microscopy to visualize encapsulation of the prodrug within the particle (Fig. 1E).

**TABLE 1:** Encapsulation efficiency of TB drugs in GLPs

Drug	Drug:GLP weight ratio	Encapsulation efficiency
Isoniazid prodrug	0.15–450 µg INH/mg GLP	>80%
Rifabutin + tannic acid	0.3–2.5 µg RFB/mg GLP at 26:1 tannic acid:RFB molar ratio	>80%
Clofazimine	6.25–800 µg CFZ/mg GLP	>75%
Clofazimine + N-dodecylimidazole	6.25–800 µg CFZ/mg GLP C_12_Im:CFZ weight ratio of 1:1	>75%
Linezolid prodrug	200–800 µg LZD/mg GLP	>70%

The Schiff base linker used in this prodrug was designed to undergo faster hydrolysis at acidic pH, GLP INH-C_12_ samples were evaluated for kinetics of INH release from the particles at pH 7 or pH 5, which mimics the change in pH upon delivery to the lysosome after phagocytosis (33). The prodrug was stable in neutral pH with <30% INH drug released from the particles after 72 h. The samples exhibited faster hydrolysis at pH 5 with ~50% of the drug released in <12 h and almost complete drug release at 72 h (Fig. 1F). These release assays were carried out at a concentration of 50 µg INH/mL, which is well below the maximum solubility of INH in water (140 mg/mL). Thus, the prodrug formulation allowed for efficient, stable encapsulation and pH-dependent release.

Rather than an acid-labile linker, a prodrug of LZD was synthesized by reaction of LZD with dodecylsulfenyl chloride to yield a compound with a sulfenamide linker that releases LZD upon reaction of the prodrug with glutathione, which mimics the reductive environment encountered upon maturation of endosomal compartments (32, 33) (Fig. 1B). The LZD prodrug was encapsulated in GLPs following a similar procedure as the INH prodrug (Fig. 1A). With this method, >70% of the LZD prodrug was encapsulated in GLPs (Fig. 1G; Table 1). Nile red staining of the hydrophobic C_12_ tail of the prodrug confirmed payload encapsulation inside the hollow cavity of GLPs (Fig. 1H). Samples of GLP LZD prodrug were evaluated for payload release at pH 7 and pH 5 with and without the addition of 10 mM glutathione. The drug remained stably encapsulated after 3 and 24 h incubation at both pH values in the absence of glutathione (Fig. 1I). These drug release assays were carried out at a concentration of 500 µg LZD/mL (maximum solubility of LZD in water is 3 mg/mL). The drug was released in 3 h upon the addition of glutathione, and the efficiency of release was maximized at pH 5 where the glutathione reaction with the sulfenamide linker is expected to be more efficient.

### GLP encapsulation of water-insoluble TB drugs

Our data with prodrugs indicated that hydrophobic compounds are stably encapsulated in GLPs, and their release can be controlled via the hydrolysis of the prodrug. To control the release process of intrinsically hydrophobic drugs we investigated co-loading the drug with excipients that either increase stability (e.g., co-loading a hydrophobic drug with a fatty acid that is solid at room temperature) or facilitate release (e.g.*,* co-loading the drug with a surfactant). Two different encapsulation approaches were developed for the hydrophobic drugs, rifabutin (RFB) and clofazimine (CFZ).

**Fig 2:**
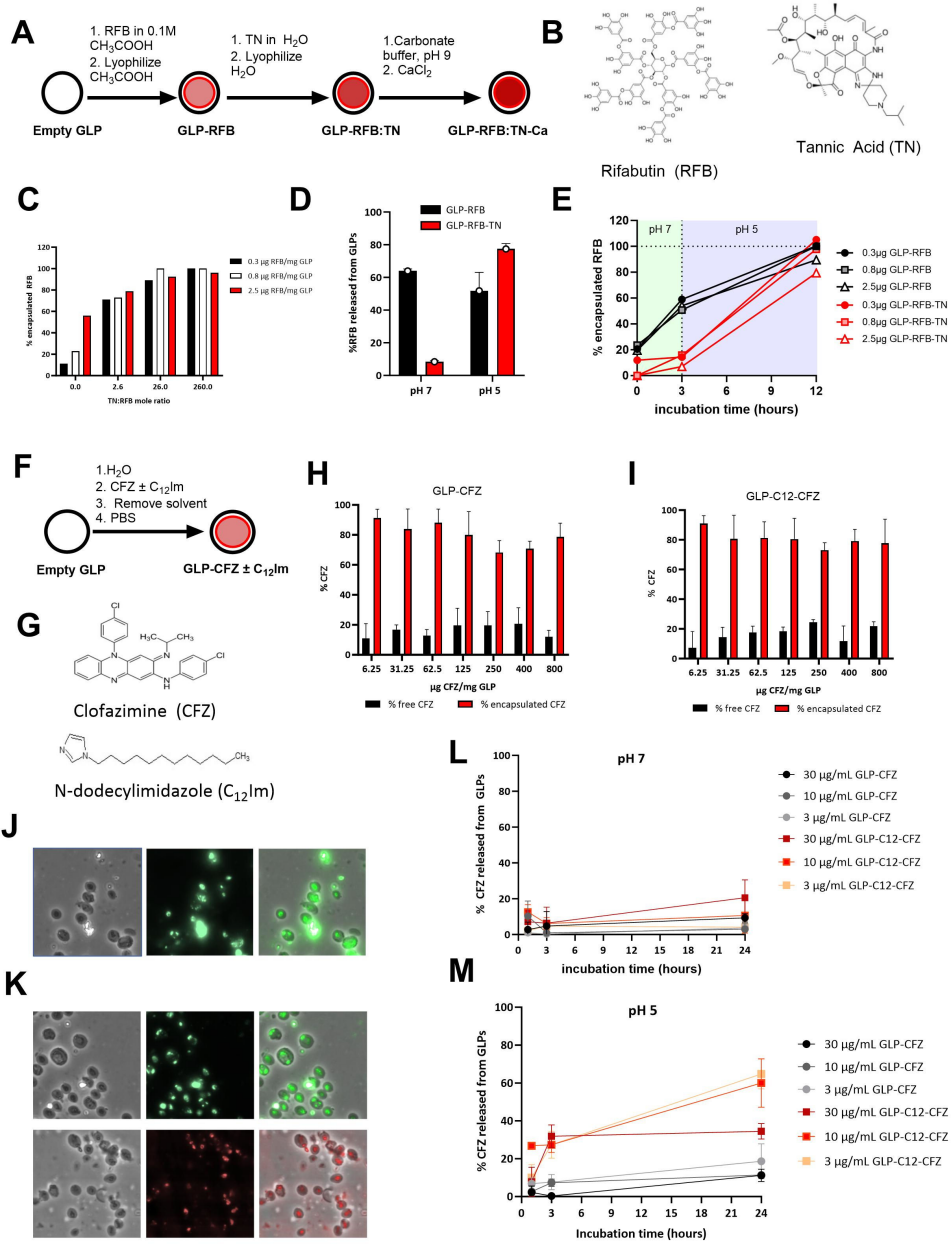
Excipients allow for encapsulation, retention, and controlled release of RFB and CFZ. **(A**) Schematic of RFB loading in GLPs by crosslinking with tannic acid (TN)-calcium chloride. (**B**) RFB and TN chemical structures. (**C**) RFB encapsulation efficiency in GLPs using TN at molar ratios of TN:RFB from 0 to 260 measured by UV/Vis spectroscopy. (**D**) RFB release from GLPs samples diluted 200 µg RFB/mL in 50 mM phosphate buffer (pH 7) or 50 mM acetate buffer (pH 5) for 24 h. (**E**) RFB release from GLP samples diluted to 100 µg RFB/mL in 50 mM phosphate buffer from t = 0 to t = 3 h and in 50 mM acetate buffer (pH 5) from t = 3 h to t = 12 h. Data shown correspond to GLP RFB samples prepared at 2.5 µg RFB/mg GLP. (**F**) Schematic of CFZ and N-dodecylimidazole (C_12_Im/surfactant) loading in GLPs. (**G**) Chemical structures of CFZ and N-dodecylimidazole. (**H-I**) Encapsulation efficiency of CFZ (**H**) and CFZ- C_12_Im samples (1:1 CFZ: C_12_Im weight ratio) (**I**) in GLPs. (**J**) First panel: brightfield images of GLP-CFZ, second panel: fluorescent images of GLP-CFZ in FITC channel exposure time 500 ms, third panel: merged images. (**K**) First panel: brightfield images of GLP- C_12_Im -CFZ, second panels: fluorescent images of GLP- C_12_Im -CFZ in FITC channel exposure time 500 ms (top) and Nile red staining exposure time 50 ms in rhodamine channel (bottom), third panel: merged images. To avoid interference between CFZ and Nile red fluorescence, different samples of GLP- C_12_Im -CFZ were used for imaging of these two compounds. (L-M) Measurement of CFZ release from GLP-CFZ and GLP- C_12_Im -CFZ samples at (**L**) pH 7 and (**M**) pH 5. Data shown correspond to GLP- C_12_Im -CFZ prepared at 250 µg CFZ/mg GLP.

RFB is slightly soluble in water (200 µg/mL) and therefore difficult to stably encapsulate on its own. Instead, RFB was loaded in GLPs using an aqueous solution of the drug, followed by an aqueous solution of tannic acid (TN). The tannic acid and RFB can be trapped inside GLPs by crosslinking with calcium chloride (Fig. 2A and B), and this complex was expected to dissociate at low pH, facilitating controlled release. The TN:RFB molar ratio was titrated at three different RFB:GLP loading ratios to identify an optimal TN:RFB ratio to achieve >90% RFB encapsulation efficiency (Fig. 2C; Table 1). The drug was efficiently encapsulated at a TN:RFB molar ratio >26. After 24 h of incubation, RFB was spontaneously released from the loaded GLP, whereas the calcium crosslinked TN complex was stable at pH 7 with <10% RFB released (Fig. 2D). At pH 5, > 80% of the RFB was released after 12 h (Fig. 2D and E).

GLP-CFZ formulations were prepared by loading a CFZ solution in chloroform (Fig. 2F). The drug is extremely hydrophobic (solubility in water <3 µg CFZ/mL), and to improve CFZ release from GLPs, the samples were also prepared by co-loading CFZ and the surfactant, N-dodecylimidazole (C_12_Im) (Fig. 2G) (34).

The drug was efficiently encapsulated at ratios ranging from 6.25 to 800 µg CFZ/mg GLP with and without surfactant (Fig. 2H and I, respectively; Table 1). Co-encapsulation CFZ and C_12_Im was performed at CFZ:C_12_Im ratios of 1:1 as this ratio generates an insoluble mixture at pH 7 and emulsification of C_12_Im-CFZ mixture at pH 5. The encapsulation of CFZ and C_12_Im was visualized by microscopic imaging using the green autofluorescence of CFZ, and the C_12_Im encapsulated in GLP was imaged using Nile red staining of the alkyl chain (Fig. 2J and K). The samples were then evaluated for release kinetics of CFZ at pH 5 and pH 7. The drug remains encapsulated in the particles with <20% release at 24 h at pH 7 (Fig. 2L) and slowly releases at pH 5 (Fig. 2M). The addition of the acid-sensitive C_12_Im surfactant increases the release of CFZ at concentrations 10 times higher (30 µg/mL) than the maximum solubility of CFZ in water.

### GLP-encapsulated TB drugs exhibit antimycobacterial activity in infected macrophages

**Fig 3:**
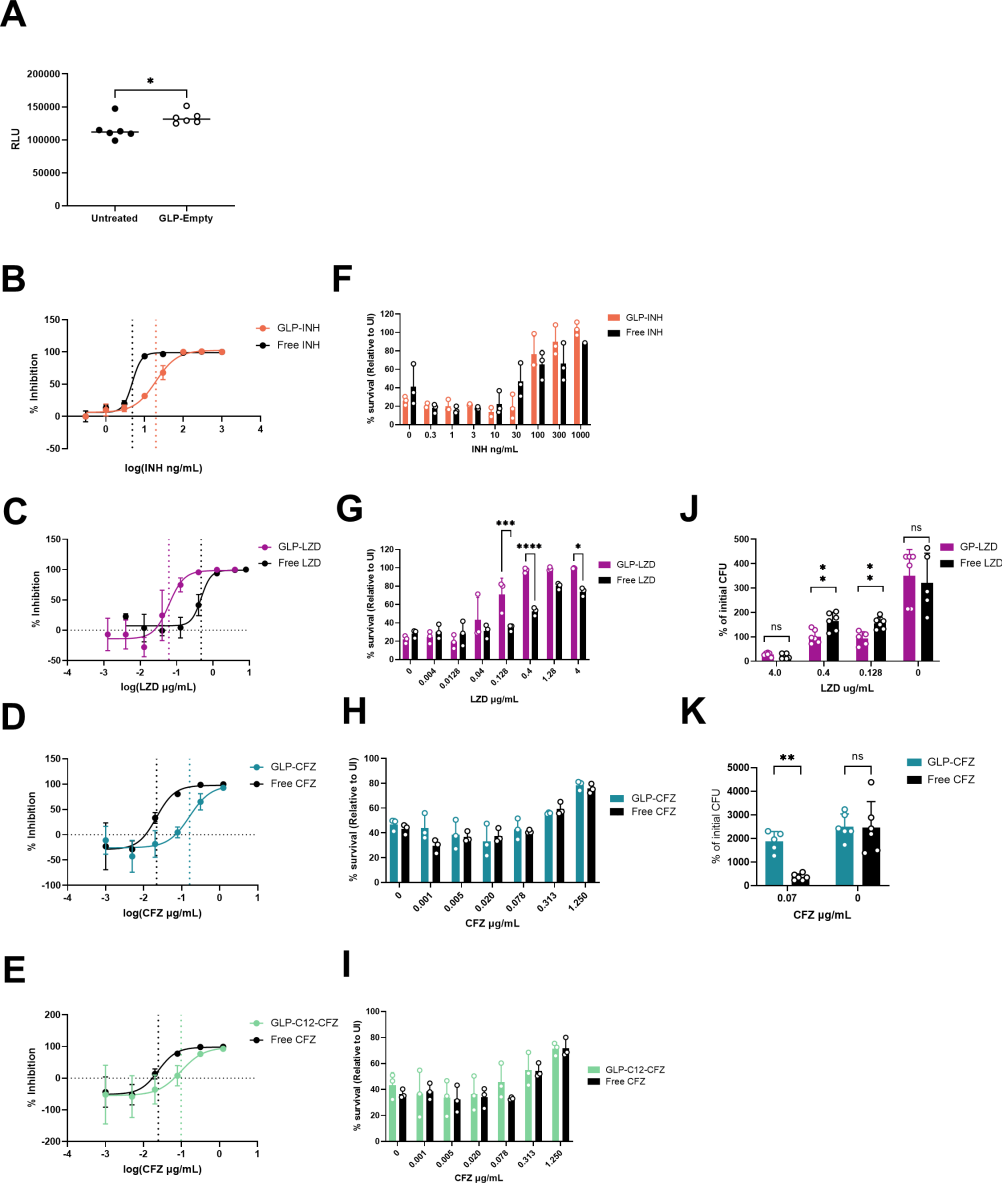
Antitubercular drugs are released from GLPs and reduce bacterial burden in infected macrophages. (A) C57BL/6J (WT) BMDMs were infected with H37Rv::Lux at an MOI = 3. BMDMs were left untreated or treated with empty GLPs for 5 days. Growth was determined by measuring bacterial luminescence after 5 days of infection. (**B-E**) % inhibition of Mtb-Lux signal by free drug and GLP encapsulated drug. BMDMs infected with H37Rv::Lux at an MOI = 3 and treated for 5 days. (**B**) INH and GLP-INH, (**C**) LZD and GLP-LZD, (**D**) CFZ and GLP-CFZ, and (**E**) CFZ and GLP- C_12_Im -CFZ. (**F-I**) Cell viability was measured 5 days post-infection using CellTiter-Glo. (**F**) INH and GLP-INH, (**G**) LZD and GLP-LZD, (**H**) CFZ and GLP-CFZ, and (**I**) CFZ and GLP- C_12_Im -CFZ. (**J**) BMDMs were infected with Mtb strain H37Rv at an MOI = 0.5. Cells were left untreated or treated with the indicated concentrations of LZD or GLP-LZD. CFU was determined 7 days post-infection. (**K**) As in panel J, infected BMDMs were left untreated or treated with free CFZ or GLP-CFZ at 0.07 µg/mL. CFU was determined 7 days post-infection. Data depicted are single experiments that are representative of at least three independent studies except for CFU assays which are representative of at least two independent studies. Statistical analysis of (**A**) was performed using an unpaired *t*-test. The remaining statistical analysis was performed using two-way ANOVA with Šidák post-test to correct for multiple comparisons. * *P*-value < 0.05, ** *P*-value < 0.01, *** *P*-value < 0.001, **** *P*-value < 0.0001.

We next tested the GLP-drug formulations in *Mtb*-infected macrophages to evaluate the effect of encapsulation on intracellular antibacterial activity. We did not move forward with the GLP-RFB constructs because the tannic acid exhibited cytotoxic effects in the initial screening (data not shown). Using a luciferase reporter strain of *Mtb* (H37Rv::Lux) to measure bacterial burden, we compared the antibacterial effects of each GLP construct to their free drug counterpart. To ensure the same number of GLPs were delivered in each condition, particles were loaded with a range of drug concentrations and delivered at a constant ratio of 10 GLP/cell. Primary bone marrow-derived macrophages (BMDMs) from C57BL/6 J mice were infected with H37Rv::Lux at an MOI of 3. Cells treated with empty GLPs had similar or slightly higher bacterial burden than untreated cells at 5 days post-infection (dpi) (Fig. 3A), indicating that GLP internalization alone does not induce antibacterial activity in macrophages.

Infected BMDMs were treated with empty GLPs, drug-loaded GLPs, or free drugs for 5 days. By day 5, all the formulations reduced bacterial burden in a dose-dependent manner, indicating that the drugs were released from the GLPs and maintained their antibacterial activity (Fig. 3B through E). For INH, prodrug modifications and encapsulation increased the MIC_50_ 4-fold compared with free drug, 4.9 ng/mL to 19.5 ng/mL (Fig. 3B). Conversely, the observed MIC_50_ of GLP-LZD (0.06 µg/mL) was almost 10-fold lower than free LZD (0.4 µg/mL) (Fig. 3C). GLP-CFZ formulations showed reduced antibacterial activity in comparison to free CFZ. The MIC_50_ of GLP-CFZ, 0.2 µg/mL, was 10-fold higher than free CFZ, 0.02 µg/mL (Fig. 3D). Similarly, the MIC_50_ of GLP-C12-CFZ, 0.1 µg/mL, was 5-fold higher than CFZ (Fig. 3E). Thus, although encapsulation quantitatively altered potency relative to free drug, all encapsulated formulations retained antibacterial activity against intracellular bacteria.

To determine if GLP treatment affected macrophage survival, we measured cell viability at 5 dpi. As expected, there was a dose-dependent increase in macrophage viability in both free drug and GLP-drug treated groups (Fig. 3F through I). In the case of GLP-INH, GLP-CFZ, and GLP-C_12_Im -CFZ, there were no significant differences in cell survival between free and GLP-drug groups across the range of tested concentrations (Fig. 3F, H and I). In contrast, GLP-LZD was significantly more effective than the free drug in maintaining macrophage viability, which is consistent with the increased activity of the encapsulated LZD in the Mtb::Lux assay (Fig. 3G).

To verify that the Mtb-Lux reporter assay and macrophage survival reflected bacterial viability, we measured colony forming units (CFU) 7 days after infection of BMDM during exposure to a range of LZD concentrations encompassing the MIC predicted by Mtb-Lux. The MIC_50_ of free LZD was comparable between Mtb::Lux and CFU assays. Also consistent with the Mtb::Lux assay, bacterial viability was significantly lower in GLP-LZD treated groups compared with free LZD groups at 0.4 µg/mL and 0.128 µg/mL (Fig. 3J). We also measured intracellular CFU in macrophages treated with GLP-CFZ and free CFZ at a dose where the difference between GLP and free drug was the greatest in the luciferase assay, 0.07 µg/mL, and found a similar effect by CFU (Fig. 3K).

In sum, the observed dose-dependent inhibition of bacterial growth demonstrated that the encapsulated biologically active payloads were released from all the formulations. In the case of INH and CFZ, delivery via GLPs quantitatively decreased antibacterial activity, but the degree of reduction did not impact overall macrophage viability at 5 dpi. GLP-mediated delivery of LZD enhanced its antibacterial effects in infected macrophages that corresponded to an increase in macrophage viability. These results demonstrated the functionality of these GLP constructs in *Mtb*-infected macrophages.

### GLP-encapsulated clofazimine reduces bacterial burden in infected mice

**Fig 4:**
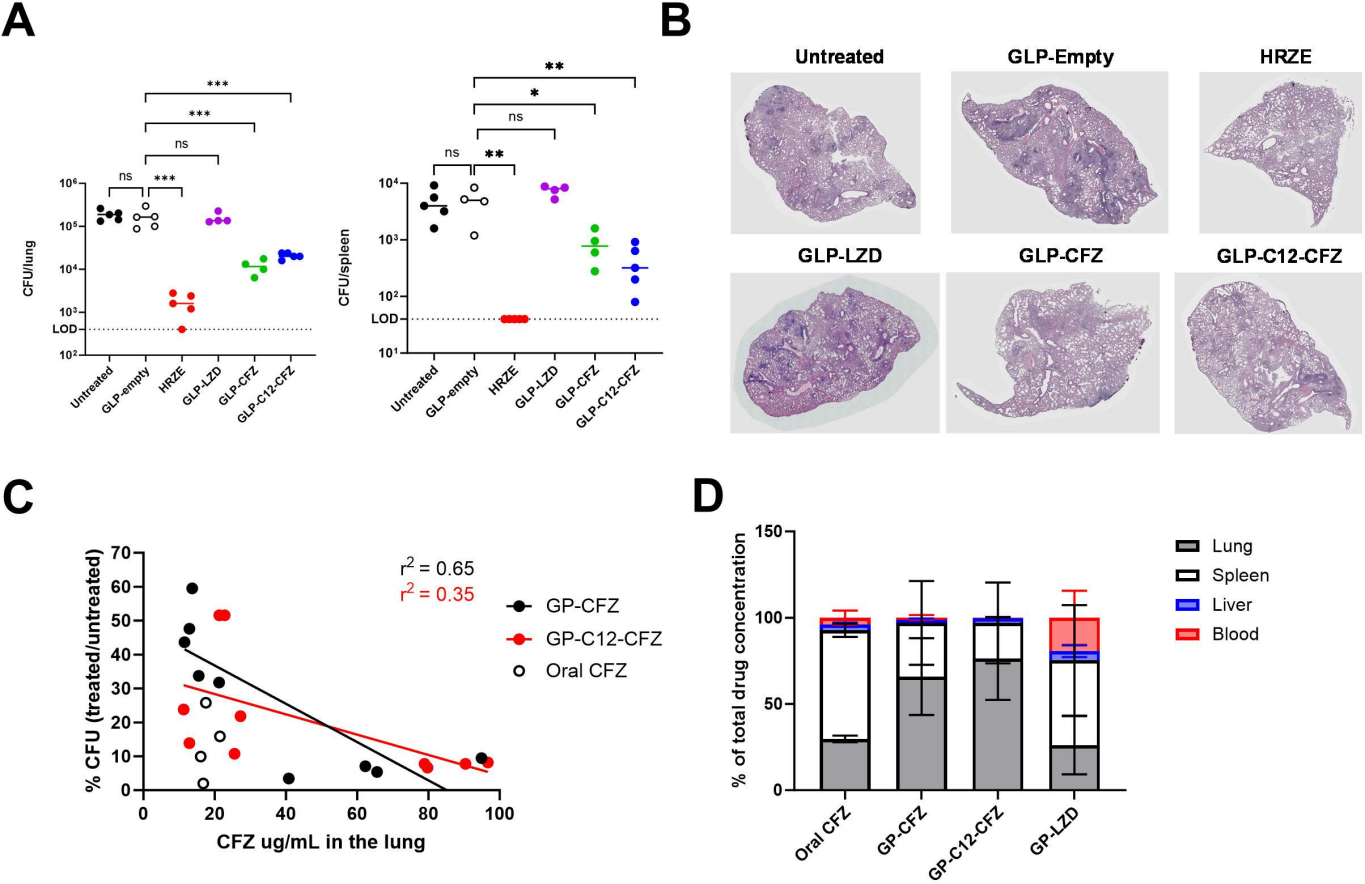
Intranasal delivery of CFZ reduces bacterial burden in mice and concentrates the drug in the lung. C57BL/6J were aerosol infected with Mtb strain H37Rv (~200 CFU per mouse). On day 21 post-infection, mice were left untreated or treated intranasally with empty-GLPs, GLP-LZD, GLP-CFZ, and GLP- C_12_Im -CFZ three times per week (800 µg drug/mg GLP) for a total of 10 treatments. Control mice were treated with HRZE in the drinking water. (**A**) Lung and spleen were harvested on day 45 post-infection, and CFU were enumerated. (**B**) Representative H&E images from lung sections were collected at the end of the experiment. (**C**) Plot of CFU (normalized to untreated) relative to the concentration of CFZ µg/mL measured in lung homogenate using HPLC across two independent experiments. (**D**) Proportion of drug recovered from lung, spleen, liver, and blood relative to total recovered drug across two independent experiments. Statistical analysis of A was performed using a one-way ANOVA with Dunnet’s post-test for multiple comparisons. * *P*-value < 0.05, ** *P*-value < 0.01, *** *P*-value < 0.001, **** *P*-value < 0.0001.

Given their importance as second-line TB therapeutics and their propensity to cause systemic side effects, we tested the GLP CFZ and LZD formulations via intranasal delivery to the lung (35, 36). Mice were infected with H37Rv, and the infection was allowed to proceed without treatment for 3 weeks. Mice were divided into six treatment groups: GLP-LZD, GLP-CFZ, GLP-C_12_Im-CFZ, GLP-Empty, oral HRZE (consisting of isoniazid, rifampicin, pyrazinamide, and ethambutol), and untreated. GLP treatments were delivered intranasally 3× per week and HRZE was administered *ad libitum* in the drinking water as a positive control for bacterial reduction. After 3 weeks of treatment, the lungs and spleens were harvested from the mice, and CFU were enumerated. There was no difference between lung or spleen CFU in mice treated with empty GLPs and untreated indicating that GLPs alone did not affect bacterial burden (Fig. 4A). Histopathology of the lungs revealed focal leukocyte infiltrates typical of murine TB, and no discernable difference between untreated animals and those treated with empty GLP (Fig. 4B). We observed no CFU reduction in the lungs or the spleens of GLP-LZD-treated mice in comparison to untreated. In contrast, both GLP-CFZ and GLP-C_12_Im-CFZ treatments significantly reduced bacterial burden in lungs and spleen by approximately 1-log in comparison to untreated mice. Oral HRZE was the most effective treatment. The relative degree of lung histopathology was consistent with the bacterial burden observed in each group.

We hypothesized that GLP encapsulation and delivery directly to the lungs would increase the concentration of drug in the lungs in comparison to systemic delivery. To test this with CFZ, groups of mice were infected for 3 weeks and then were either left untreated or treated with oral CFZ, GLP-CFZ, GLP- C_12_Im -CFZ, GLP-empty, or HRZE. Oral clofazimine was delivered daily via oral gavage in accordance with previous literature (19). GLPs were delivered 3× per week intranasally, and HRZE was available in the drinking water for 3 weeks. Drug was extracted from lung homogenate and quantified using HPLC. Across multiple infection studies, we found that the GLP formulations achieved a wide range of CFZ concentrations in the lungs (11.3–96.7 µg/mL) (Fig. 4C). For both GLP CFZ formulations lung CFU was inversely correlated with the concentration of CFZ. Oral delivery of CFZ resulted in consistently lower concentrations of drug in the lungs (16.0–21.4 µg/mL) and relatively low CFU (Fig. 4C).

To determine if direct delivery to the lungs limited systemic drug exposure and increased the proportion of drug in the lungs, drug concentration was assessed in blood, liver, spleen, and lung samples 24 h after the last dose. The majority of CFZ was detected in the lungs in mice treated with GLP-CFZ or GLP- C_12_Im -CFZ, whereas the majority of CFZ was found in the spleens of mice treated with oral CFZ (Fig. 4D). Consistent with the lack of antibacterial effect for GLP-LZD, we found that LZD was not concentrated in the lungs in mice treated with this formulation; the majority was detected in the spleen and the blood (Fig. 4D).

Overall, GLP encapsulation of CFZ and LZD enabled direct delivery to the lungs. We conclude that GLP-LZD did not reduce bacterial burden in mice likely because the drug was not retained at the site of infection. In the case of GLP-CFZ and GLP- C_12_Im -CFZ, direct delivery to the lungs significantly reduced bacterial burden in mice and concentrated the drug in the infected organ, thereby reducing the systemic exposure that results in skin pigmentation and discoloration.

## DISCUSSION

Inhaled tobramycin is an effective treatment for pulmonary *Pseudomonas aeruginosa* infections in cystic fibrosis patients, which highlights the utility of aerosol antibiotic therapies (37, 38). Other inhalable formulations of aminoglycosides and other antimycobacterial drugs have shown promise in treating mycobacterial infections in humans and animal models (11, 12, 18, 39–41). Aminoglycosides are particularly attractive for aerosol delivery because they are retained in lung tissue (42). However, these pharmacokinetic characteristics vary greatly across different types of antibiotics meaning that different formulations are likely required to deliver the chemically diverse collection of TB drugs. In this study, we aimed to test whether glucan lipid particles could serve as a flexible inhalable delivery platform for the controlled release of diverse drugs in the lung. We identified the strengths of the GLP system as well as challenges related to individual drug pharmacology.

GLPs preserve the characteristics of glucan particles (GPs) that make them attractive for TB drug delivery while expanding the range of compounds that are compatible with encapsulation. Previously, the utility of GPs for small molecule delivery was limited because (i) many small molecules have a neutral charge and are not easily trapped using previously described polyplex and layer-by-layer approaches (25, 43, 44), (ii) highly water-soluble small molecules are not retained in GPs, and (iii) water-insoluble small molecules are loaded inefficiently. We present multiple strategies for GLP encapsulation of a wide variety of hydrophobic and hydrophilic drugs, either by the generation of prodrugs or the co-encapsulation of excipients. By combining these approaches, we believe that GLPs are pliable enough to accommodate co-encapsulation of multiple drugs. This approach would ensure sustained combination therapy, which is particularly important for eradicating mycobacterial infections and suppressing the emergence of drug-resistant strains.

We were most successful with the GLP CFZ formulations. When loaded alone or in combination with surfactant, we were able to achieve stable encapsulation across more than a 3 log_10_ range of drug concentrations. After 3 weeks of GLP CFZ treatment, both formulations reduced bacterial burden in *Mtb*-infected mice. Like other inhalable formulations of clofazimine, GLP delivery concentrated CFZ in the lungs in comparison to oral delivery (19, 45, 46). The pharmacokinetic properties of CFZ are amenable to aerosol delivery. This compound is sequestered in lipid- and macrophage-rich regions like the lungs, likely contributing to its retention in the lungs after aerosol delivery (19, 46–48). Although the results of this *in vivo* study were promising, we noted that oral CFZ tended to reduce bacterial burden at lower lung concentrations than GLP CFZ (Fig. 4C). We hypothesize that the discrepancy in antibacterial activity is due to different lesional pharmacokinetics of systemically delivered versus GLP-delivered CFZ. Oral administration likely allows for intravascular delivery and potentially better lesion penetration than the GLP-delivered CFZ. Regardless, inhaled GLP-CFZ produced a similar bactericidal effect as oral delivery while reducing the systemic exposure of this drug that leads to stigmatizing skin discoloration (35, 49), achieving a primary goal of this approach. We note that the large majority of bacteria remain intracellular in the mouse model used in these studies, in contrast to acellular niches occupied by Mtb in the necrotic lesions found in humans. Although GLPs are unlikely to deliver their cargo directly to these necrotic sites, concentrating drug release in the directly adjacent macrophages could still increase and/or prolong local drug exposure. Additional investigation of local drug pharmacology in other animal models will be necessary to test this hypothesis.

Although we had success with encapsulating and delivering CFZ, we had less success with other drugs. For example, the GLP-RFB formulation was limited by the toxicity of the tannic acid excipient and requires further modifications before it will be suitable for *ex vivo* testing. Conversely, encapsulation of LZD increased the potency of this drug in *ex vivo* macrophage cultures, but this formulation was not active in mice. We were not able to detect the LZD prodrug in the lung (data not shown), indicating that the free drug was fully released from the GLP. However, unlike CFZ, which is retained in the lung, LZD is more soluble, has a much shorter tissue half-life (50), and was found predominantly in extrapulmonary sites. Improving the activity of GLP-LZD may require altering the formulation to further slow drug release to prolong drug exposure.

In summary, we present these experiments as a proof of concept for controlled aerosol delivery of antimycobacterial drugs in GLPs. This work demonstrated that preserving non-saponifiable lipids in the membrane of GLPs makes them better suited than GPs for encapsulation and retention of water-insoluble small molecules. We were able to develop approaches for loading water-soluble and water-insoluble drugs into GLPs and controlling their release. However, intranasal administration directly to the site of infection was not sufficient to overcome some of the limiting properties inherent to each compound. Inhalable drugs have the potential to improve TB treatment by decreasing dose frequency, increasing adherence to avoid painful injections, and improving safety by limiting systemic exposure. Beyond TB, inhalable antibiotic delivery platforms have implications for treating non-tuberculous mycobacterial infections that are even more difficult to treat (51). A malleable inhalable drug platform that maximizes the potential of existing drugs could rapidly improve the treatment of mycobacterial infections.

## MATERIALS AND METHODS

### Reagents for GLP drug encapsulation

Yeast particles were obtained from Biorigin (Louisville, KY); clofazimine from Sigma Aldrich (Waltham, MA); rifabutin, 1-dodecylimidazole, dodecanal, isoniazid, and linezolid from Thermo Fisher Scientific (Waltham, MA); dodecanal from Alfa Aesar (Haverhill, MA); and dodecyldisulfide from TCI (Fremont, CA). All other reagents and solvents for the synthesis of prodrugs and particles, and HPLC grade solvents were purchased from Thermo Fisher Scientific.

### Glucan particle (GP) and glucan lipid particle (GLP) preparation

The preparation of GPs follows the same procedure previously described (43). The glucan lipid particles were prepared in the following manner. Yeast particles (*Saccharomyces cerevisae*) are suspended in 1 L of 1M NaOH and heated to 85°C . The cell suspension is stirred vigorously for 1 h at this temperature. The insoluble material containing the yeast cell walls is recovered by centrifugation (14,000 rpm, 30’). The pellet is then suspended in 1M NaOH, heated, and stirred vigorously for 1 h at 85°C. The suspension is allowed to cool to room temperature (20–25°C), and the extraction is continued for a further 16 h. The insoluble residue is recovered by centrifugation. This insoluble residue is finally extracted in water and brought to pH 4.5 with HCl. The insoluble residue is recovered by centrifugation and washed three times with water. The resulting slurry is placed in glass trays and dried under reduced pressure to produce a fine light yellow powder. GLPs were sterilized by autoclaving dry GLP samples in a closed glass container at 120°C for 30 min. Sterile GLPs were used for the synthesis of fluorescent particles and the loading of TB drugs.

### Mice

C57BL/6 J mice were purchased from the Jackson Laboratory. Housing and experimentation were in accordance with the guidelines set forth by the Department of Animal Medicine of University of Massachusetts Chan Medical School and the Institutional Animal Care and Use Committee. Animals used for experimentation were between 8 and 10 weeks old.

### Bacterial growth and strain generation

*Mycobacterium tuberculosis* strains were cultured at 37°C in complete Middlebrook 7H9 medium containing oleic acid-albumin-dextrose-catalase (OADC, Becton, Dickinson), 0.2% glycerol, and 0.05% Tween80 or 0.02% Tyloxapol. Kanamycin and zeocin were added at 25 µg/mL. The *Mtb* luciferase reporter strain was generated by transforming wild type H37Rv with the Tweety integrating plasmid that contains the luciferase gene. Luciferase activity was confirmed in the H37Rv-Lux::Zeo by measuring relative luminescence in serial dilutions of the strain in comparison to wildtype H37Rv.

### Macrophage infections

Bone marrow-derived macrophages (BMDMs) were isolated from C57BL/6 J mice by culturing bone marrow cells in DMEM supplemented with 20% conditioned medium from L929, 10% FBS, 2 mM L-glutamine, and 1 mM sodium pyruvate for 7 days. BMDMs were seeded in 96-well white, opaque, tissue culture-treated plates (Corning 3917) and left overnight. The next day, cells were infected with *Mtb* at an MOI of 3 or 0.5 for the luciferase report assay or CFU assay, respectively. After 4 h incubation, macrophages were washed twice with PBS to remove extracellular bacteria and incubated in a fresh complete medium with or without drugs (encapsulated or free). GLPs were delivered at an MOI of 10. GLP and free drugs were diluted in cell media, DMEM supplemented with 10% FBS. Relative luminescence was measured using the Agilent BioTek Synergy H4 Hybrid Microplate Reader on day 5 post-infection. For intracellular CFU, cells were lysed with 1% Saponin/PBS 7 days post-infection and then plated on Middlebrook 7H10 agar (Fisher Scientific Cat. No. B12351) supplemented with 4 g/L activated charcoal (Sigma C9157), glycerol, and OADC and containing kanamycin (25 µg/mL) in serial dilutions. CFUs were counted after 4 weeks of incubation at 37°C.

### Macrophage viability assay

Macrophage viability was measured 5 days post-infection using CellTiter-Glo 2.0 Cell Viability Assay according to manufacture instructions. Luminescence was measured using the Agilent BioTek Synergy H4 Hybrid Microplate Reader.

#### Mouse infections

Prior to infection, BcRv::Kan was resuspended and sonicated in PBS containing 0.05% Tween80. Mice were inoculated with approximately 200 CFU via the respiratory route using an aerosol generation device (Glas-Col). Mice were divided into treatment groups 21 days post-infection. In GLP treatment groups, each mouse received 25 µL of GLPs (500 µg of CFZ or LZD per dose or 800 µg drug/mg GLP, 25 mg GLP/mL) intranasally three times per week for a total of 10 treatments. For the oral clofazimine group, mice received 20 mg/kg CFZ suspended in 200 µL of a 0.5% agarose solution daily (5 days per week) for 3 weeks. For the mice receiving HRZE, antibiotics were administered via drinking water at the following concentrations: 0.1  g/L isoniazid (Sigma), 1.2  g/L ethambutol (Sigma), 0.1  g/L rifampin (Sigma), and 1.5  g/L pyrazinamide (Sigma). Antibiotic water was changed 3 times per week. At 42 days post-infection, mice were sacrificed. Blood samples were taken via cardiac puncture. Liver, spleen, and lung sections were collected for measuring tissue drug concentration and histopathology. Lung and spleen CFU were determined by plating serial dilutions of homogenate on Middlebrook 7H10 agar (Fisher Scientific Cat. No. B12351) supplemented with 4 g/L activated charcoal (sigma C9157), glycerol, and OADC and containing kanamycin (25 µg/mL). CFUs were counted after 4 weeks of incubation at 37°C.

### Drug tissue extraction and quantification

#### CFZ extraction from tissues

CFZ was extracted from mice tissues following a procedure reported by Borner (52). Tissue samples were incubated in 2 mL of 90% methanol and 10% acetic acid for 3 h at 25°C at 300 rpm. The samples were centrifuged, and the supernatant was diluted with 3 mL of acetonitrile and incubated 1 h at 25°C to precipitate protein. The samples were centrifuged, the supernatant was collected, and the solvent was evaporated. The dry samples were dissolved in HPLC mobile phase and quantified by HPLC. The tissues were extracted three times.

#### LZD extraction from tissues

LZD was extracted from mice tissues by adapting a literature procedure (53). Tissue samples were incubated in 2 mL PBS for 5 h. The samples were centrifuged (3,000 rpm, 10’), the supernatant was collected, diluted with 2 mL of acetonitrile, incubated 1 h to precipitate protein, and centrifuged, and then, the supernatant was collected, and the solvent was removed. The dry samples were dissolved in HPLC mobile phase to quantify extracted LZD. The tissue samples were extracted twice in PBS. Then, tissue samples were incubated overnight at 37°C in 2 mL of 50 mM acetate buffer (pH 5) containing 10 mM GSH to extract LZD still present in the tissues as LZD prodrug. The samples were centrifuged, and the supernatant was collected and processed to quantify LZD by HPLC.

### Synthesis of INH prodrug

A Schiff base prodrug of INH was synthesized following experimental methods described for similar INH prodrugs (31, 54). A mixture of INH (10 mmol) in methanol (15 mL) was heated at reflux temperature to completely dissolve the drug. A solution of dodecanal (10 mmoL) in methanol (3 mL) was slowly added to the INH solution, and the mixture was heated at reflux for 1.5 h. The reaction mixture was slowly cooled to room temperature and stirred overnight. The reaction product was filtered, washed with cold (4°C) methanol, and dried under vaccuum (80% yield, m.p. 85–87°C, ^1^H-NMR (CDCl_3_) 400 MHz, δ ppm: 8.7 (2H, d); 7.7 (3H, m); 2.3 (2H, m); 1.2–1.5 (18 H, m); and 0.8 (3H, t)

### Synthesis of LZD prodrug

The LZD prodrug was synthesized following a reported literature procedure for the synthesis of sulfenamide prodrugs of LZD (32, 55). Didodecylsulfide (0.6 mmol) in tetrahydrofuran (THF, 25 mL) was cooled at 4°C; sulfuryl chloride (2.4 mmol) was added; and the mixture was stirred at 4°C for 2 h to form dodecylsulfenyl chloride. Linezolid (0.9 mmol) was dissolved in THC (30 mL), cooled at 4°C; triethylamine (1.2 mmol) was added to the LZD solution, and this reagent solution was added to the mixture of dodecylsulfenyl chloride in THF. The reaction was allowed to proceed at 4°C for 1 h, and then, the ice bath was removed to allow the reaction mixture to reach room temperature (23°C) and was stirred overnight. The THF solution was filtered, the solvent was removed under reduced pressure, and the residue was purified by column chromatography (hexane:ethyl acetate:methanol 20:40:40) to obtain the LZD prodrug (30% yield, ^1^H-NMR (CDCl_3_) 400 MHz, δ ppm: 7.5 (1H, d); 7.1 (1H, s); 6.9 (1H, d); 4.8 (1H, m); 4.1 (2H, d); 3.6–3.8 (6H, m); 3.1 (4H, t); 2.7 (2H, t); 2.0 (3H, s); 1.8 (2H, q); 1.2–1.4 (16H, m); and 0.9 (3H, t))

### GLP loading of INH prodrug

Dry GLPs were mixed with 0.5 µL water per mg GLP. Then, INH prodrug was absorbed into GLPs by swelling the particles with a solution of INH prodrug in chloroform (4 µL/mg GLP). The samples were incubated at room temperature for 18–24 h to complete loading. The GLP INH prodrug was lyophilized. To maximize loading of INH prodrug into GLPs, the dry GLP-INH samples were hydrated with 0.5 µL water per mg GLP, mixed with 2 µL CHCl_3_/mg GLP, incubated overnight at room temperature, frozen, and lyophilized. The lyophilized GLP INH prodrug samples were suspended in PBS at a concentration of 10 mg GLP/mL and stored at −20°C .

### GLP loading of LZD prodrug

Dry GLPs were mixed with 1 µL water per mg GLP. Then, LZD prodrug was absorbed into GLPs by swelling the particles with a solution of LZD prodrug in dimethylsulfoxide (DMSO, 4 µL/mg GLP). The samples were incubated at room temperature for 18–24 h to complete loading. The GLP LZD prodrug was then lyophilized, and the loading process was repeated until target concentrations of encapsulated LZD were achieved. To maximize loading of LZD prodrug into GLPs, the dry GLP-CFZ samples were hydrated with 0.5 µL water per mg GLP, mixed with 2 µL DMSO/mg GLP, incubated overnight at room temperature, frozen, and lyophilized. The lyophilized GLP LZD prodrug samples were suspended in PBS at a concentration of 10 mg GLP/mL and stored at −20°C.

### GLP loading of RFB

Dry GLPs were mixed with a rifabutin solution in 0.1 M acetic acid (5 µL/mg GLP), incubated for 30 min at room temperature, frozen, and lyophilized. To maximize drug loading into GLPs, the dry GLP RFB samples were mixed with 2.5 µL 0.1 M acetic acid/mg GLP, incubated for 30 min at room temperature, frozen, and lyophilized. Then the mixing, incubation, freezing, and lyophilization steps were repeated with: (i) a solution of tannic acid in water (5 µL/mg GLP) (ii), water (2.5 µL/mg GLP) (iii), sodium carbonate buffer pH 9 (5 µL/mg GLP), and (iv) water (2.5 µL/mg GLP). The samples were finally mixed with 2.5 M CaCl_2_ solution adjusted to pH 7 (5 µL/mg GLP), incubated 30 min at room temperature and then diluted with PBS at a concentartions of 10 mg GLP/mL and stored at −20°C.

### GLP loading of CFZ with and without N-dodecylimidazole (C_12_Im) surfactant

Dry GLPs were mixed with 0.5 µL water per mg GLP. Then, CFZ was absorbed into GLPs by swelling the particles with a solution of CFZ in chlorofrom (5 µL/mg GLP) or a solution of CFZ and C_12_Im in chloroform (CFZ:C_12_Im weight ratio of 1:1). The samples were incubated at room temperature for 18–24 h to complete loading. The GLP CFZ was then lyophilized, and the loading process was repeated until target concentrations of encapsulated CFZ and C_12_Im were achieved. To maximize loading of drug into GLPs, the dry GLP-CFZ samples were hydrated with 0.5 µL water per mg GLP, mixed with 2.5 µL CHCl_3_/mg GLP, incubated overnight at room temperature, frozen, and lyophilized. The lyophilized GLP CFZ ± C_12_Im samples were mixed with PBS (5 µL/mg GLP), incubated 2 h at room temperature, and then, PBS was added to adjust the sample to a final concentration of 10 mg GLP/mL.

### GLP microscopy

Encapsulation of INH, LZD, and CFZ was qualitatively assessed by fluorescence microscopy using an Olympus BX60 upright compound fluorescent microscope. GLP-INH, GLP-LZD, and GLP-C_12_Im-CFZ were stained with Nile red and measured in the rhodamine channel with an exposure time of 50 ms unless otherwise indicated. CFZ autofluorescence was measured in the FITC channel with an exposure time of 500 ms unless otherwise indicated.

### Encapsulation efficiency assays

All GLP samples were thawed and suspended by tip sonication. The remaining preparation was unique for each construct.

### GLP-INH encapsulation efficiency

The samples were centrifuged, and the supernatant was collected to quantify free INH. An aliquot of each supernatant was analyzed by HPLC to quantify free and encapsulated INH and INH prodrugs. A second aliquot of each supernatant was lyophilized, dissolved in 0.1 M citrate buffer (pH 3), incubated overnight at room temperature, and analyzed by HPLC. Samples were analyzed by HPLC using a Beckman Coulter operated with 32 Karat software version 7.0 (Beckman Coulter, Inc, Brea, CA, USA), using a Zorbax Eclipse XD8 C8 column (5 µm, 4.6 × 150 mm) with methanol-water (90:10) as mobile phase, at a flow rate of 0.5 mL/min, an injection volume of 10 µL, and INH detection by absorbance measurement at 254 nm (INH retention time of 3.2 min, INH prodrug retention time of 6.3 min).

### GLP-LZD encapsulation efficiency

GLP LZD prodrug samples were thawed, suspended by tip sonication to aliquot samples containing 30 µg LZD. The samples were centrifuged, and the supernatant was collected to quantify free LZD. Both the supernatant and pellets were incubated in 50 mM acetate buffer containing 10 mM glutathione (GSH) overnight at 37°C. The pellet fractions were centrifuged to collect the supernatant containing the extracted drug. Samples were diluted 1:1 in HPLC mobile phase and analyzed by HPLC using a Zorbax Eclipse XD8 C8 column (5 µm, 4.6 × 150 mm) with acetonitrile-water (80:20) as mobile phase, a flow rate of 0.8 mL/min, an injection volume of 30 µL, and LZD detection by absorbance measurement at 251 nm (LZD retention time of 8.8 minutes).

### GLP-RFB encapsulation efficiency

GLP RFB samples were thawed and suspended by tip sonication to aliquot samples containing 50 µg RFB. The samples were centrifuged, and the supernatant was collected to quantify free RFB. The pellets were resuspended in 0.1 M acetate buffer at a concentration of 100 µg RFB/mL, and incubated overnight at room temperature and centrifuged, and the supernatant was collected to quantify extracted RFB prodrug. A second extraction was done by incubating the GLP RFB pellets overnight in a mixture of 10% water and 90% DMSO. The supernatants containing extracted RFB were lyophilized, and the residue was dissolved in 200 µL of DMSO. The drug was quantified by absorbance measurement at 510 nm, and the concentrations were calculated using a calibration curve of RFB standards in 100% DMSO (0–500 µg RFB/mL).

### GLP-CFZ encapsulation efficiency

The samples were lyophilized, and then, methanol was added to the dry GLP CFZ (target concentration of 250 µg CFZ/mL); the samples were centrifuged to collect supernatant (free CFZ). The pellets were resuspended in a solvent mixture of 20% water and 80% methanol and incubated overnight at room temperature and centrifuged, and the supernatant (extracted encapsulated CFZ) was collected. The supernatants containing free and encapsulated CFZ were diluted 1:1 in HPLC mobile phase and analyzed for CFZ by HPLC using an Agilent Eclipse Plus C18 column (3.5 µm, 4.5 × 150 mm) with methanol (75%) and 100 mM acetate buffer pH 5 (25%) as mobile phase, at a flow rate of 0.6 mL/min, an injection volume of 30 µL, and CFZ detection by absorbance measurement at 285 nm (CFZ retention time of 8.6 min).

### GLP drug release assays

#### INH release assay

GLP-INH prodrug samples were homogenized by tip sonication and diluted in 50 mM phosphate buffer (pH 7) or 50 mM acetate buffer (pH 5) at a concentration of 50 µg INH/mL. The samples were incubated at 37°C, and aliquots were collected at predetermined time points (up to 72 h) to quantify the amount of INH released from GLPs.

#### LZD release assay

GLP-LZD prodrug samples were homogenized by tip sonication and diluted in 50 mM acetate buffer (pH 5) with and without 10 mM GSH at a concentration of 500 µg LZD/mL. The samples were incubated at 37°C, and aliquots were collected at 1, 3, and 24 h to quantify the amount of LZD released from GLPs.

#### RFB release assay

GLP-RFB-TN samples were homogenized by tip sonication and diluted in 50 mM phosphate buffer (pH 7) at a concentration of 200 µg RFB/mL. The samples were incubated at 37°C for 3 h and centrifuged, and the supernatant was collected to quantify released RFB. The GLP RFB pellets were suspended in 50 mM acetate buffer (pH 5) at a concentration of 200 µg RFB/mL and incubated at 37°C . Aliquots were collected at predetermined time points and centrifuged, and the supernatant was collected. The amount of released RFB was quantified by absorbance measurement at 510 nm using a calibration curve of RFB standards in phosphate or acetate buffer (0–200 µg RFB/mL).

#### CFZ release assay

GLP-CFZ and GLP-C_12_Im-CFZ samples were homogenized by tip sonication and diluted in 50 mM phosphate buffer (pH 7) or 50 mM acetate buffer (pH 5) at target concentrations of 3, 10, and 30 µg CFZ/mL. The samples were incubated at 37°C, and aliquots were collected at 1, 3, and 24 h to quantify the amount of CFZ released from GLPs.
